# Supplementary material for: The causal role of circulating inflammatory markers in osteoporosis: a bidirectional Mendelian randomized study
Source: Front Immunol. 2024 Jul 18;15:1412298. doi: 10.3389/fimmu.2024.1412298 (PMC11291241; doi:10.3389/fimmu.2024.1412298)
Supplement: Supplementary file 1 [file DataSheet_1.zip › supplementary material/CIM-OSTEOPOROSIS /GCST90274815.csv_forest.pdf]

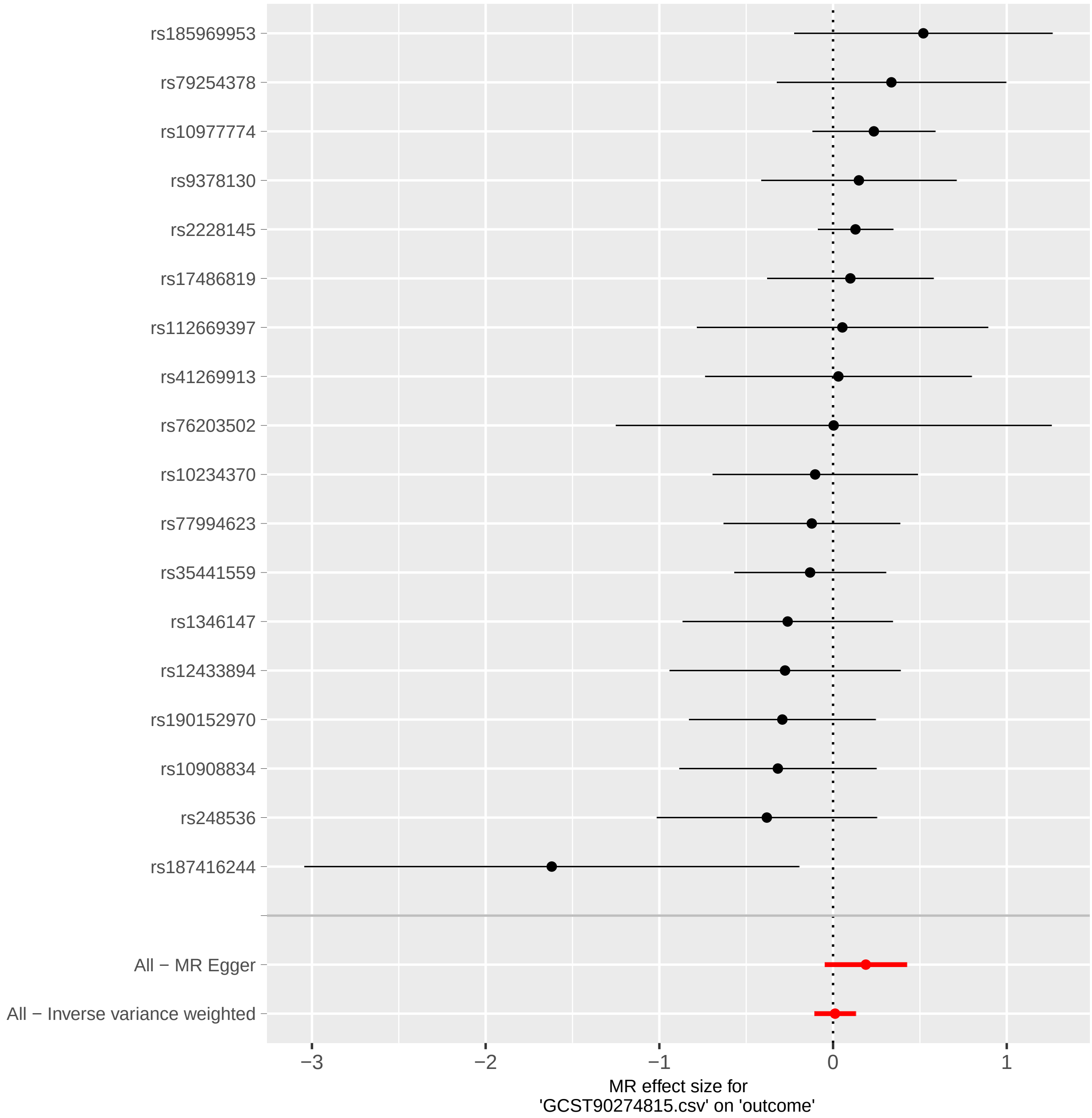
rs185969953
rs79254378
rs10977774
rs9378130
rs2228145
rs17486819
rs112669397
rs41269913
rs76203502
rs10234370
rs77994623
rs35441559
rs1346147
rs12433894
rs190152970
rs10908834
rs248536
rs187416244
All − MR Egger
All − Inverse variance weighted
−3
−2
−1
0
1
MR effect size for
'GCST90274815.csv' on 'outcome'
